# Hydrogels for Therapeutic Delivery: Current Developments and Future Directions

**DOI:** 10.3390/gels11090675

**Published:** 2025-08-23

**Authors:** Michael Arkas, Georgia Kythreoti

**Affiliations:** 1 Institute of Nanoscience Nanotechnology, NCSR “Demokritos”, Patriarchou Gregoriou Street, 15310 Athens, Greece; 2 Department of Science and Mathematics, School of Liberal Arts and Sciences, The American College of Greece, Deree, Gravias 6, 15342 Athens, Greece; gkythreoti@acg.edu

## 1. Introduction

Medical gels, in general, are regarded as promising platforms appropriate for a diversity of applications, including regenerative medicine (tissue engineering, wound healing) [1], scaffolds [2], antimicrobial and antibiofilm protection [3], hemostatics [4], lubricants [5], cartilage replacements [6], and biosensors [7], all of which are applications of paramount significance that may also promote advancement to all of the above fields reside in the field of drug delivery. The predominantly hydrogel-based targeted and/or controlled release formulations possess unique and tunable physicochemical properties: high mechanical strength accompanied by controllable biodegradation and bioadhesion. Their biocompatibility is attributed to their elastic moduli, which are comparable to those of biological tissues, and to the fact that they can mimic the extracellular matrix.

## 2. Current Developments Included in the Special Issue

### 2.1. Thermoresponsive Gels with Rosemary Essential Oil: A Novel Topical Carrier for Antimicrobial Therapy and Drug Delivery Applications

One of the most common implementations among the plethora of experimentations in the field of controlled release is thermoresponsive formulations. In one instance, Bejenaru et al. added essential oil extract from *Rosmarinus officinalis* into poly(lactic-*co*-glycolic acid) (PLGA) microparticles. In addition to the known antioxidant and anticholinesterase properties of the active ingredient [8], the composition exhibited a potent wound-healing capacity accompanied by antimicrobial properties (contribution 1).

### 2.2. Self-Assembled Peptide Hydrogels PPI45 and PPI47: Novel Drug Candidates for Staphylococcus aureus Infection Treatment

Another prophylactic scheme against skin wound infections exploits the bactericidal mechanism of defensin [9]. Hydrogels produced by two self-assembled amino acid sequences extracted from the defensin peptide PPI42, against *S. aureus* ATCC43300, exhibited efficient antibacterial protection, having minimal inhibitory concentrations between 4 and 16 µg/mL, while demonstrating minimal cytotoxicity on human epidermal cells (contribution 2).

### 2.3. Polysaccharide Hydrogels as Delivery Platforms for Natural Bioactive Molecules: From Tissue Regeneration to Infection Control

Analogous protection against microbes, paired with protracted and/or stimulus-triggered release [10] of agents promoting tissue restoration, is provided by plant-based polysaccharides. These include cellulose, dextran, hyaluronic acid, pectin, zein, pullulan, and starch (contribution 3).

### 2.4. Chitosan-Type A-Gelatin Hydrogels Used as Potential Platforms in Tissue Engineering for Drug Delivery

In addition to those mentioned above, hybrid hydrogels containing natural polymers in general, for instance, chitosan and gelatin, combine biocompatibility and biodegradability with low toxicity, which are key attributes for efficient drug delivery [11]. The latter two components, i.e., chitosan and gelatin, were employed as a host for a mainstream antibiotic (tetracycline) and achieved prolonged drug delivery for 6 h (contribution 4).

### 2.5. Tissue Regeneration and Remodeling in Rat Models After Application of Hypericum perforatum L. Extract-Loaded Bigels

The treatment of injuries and burns by composite gels containing agents that promote tissue engineering consists, in fact, of a major therapeutic outcome. A natural extract from St. John’s wort rich in hyperforin was introduced to biphasic hydrophilic-oleophilic gels [12]. Both the free compound and the analog encapsulated in “nanostructured lipid carriers” restored lesions induced in a rat skin-excision model (contribution 5).

### 2.6. Plant-Based Nanovesicular Gel Formulations Applied to Skin for Ameliorating Anti-Inflammatory Efficiency

In the majority of cases, the treatment for wounds and burns must be accompanied by gel systems, ideally based on natural products, that prevent or address inflammation [13]. Transdermal nanovesicular gel formulations possess the ability to pass through biological membranes and specifically direct the medicinal agents to the intended site via controlled release, reducing their adverse effects while increasing therapeutic effectiveness (contribution 6).

### 2.7. Integrated In Vivo and In Vitro Evaluation of a Powder-to-Hydrogel, Film-Forming Polymer Complex Base with Tissue-Protective and Microbiome-Supportive Properties

A hydrogel precursor powder is proposed for acute and chronic wound management. Combined with misoprostol and phenytoin, it accelerates wound re-epithelialization through keratinocyte migration. Microbiome studies revealed antibacterial activity against Staphylococcus and thus antibiofilm potential (contribution 7).

### 2.8. Antibiotic-Loaded Dendrimer Hydrogels in Periodontal Bone Regeneration: An In Vitro Release Feasibility Study

Teeth and bone restoration are as important as soft tissue healing and require antimicrobial screening as well. The dendrimers and the other dendritic polymers are established hosts for pharmaceutics and bioactive agents that induce appropriate cell accumulation, orientation, and organization [14]. PAMAM dendrimer, combined with polyethylene glycol diacrylate, yielded a hydrogel capable of prolonged cefazolin release, thereby facilitating effective periodontal bone regeneration (contribution 8).

### 2.9. Extremely Rapid Gelling Curcumin Silk-Tyrosine Crosslinked Hydrogels

Hydrogel precursor solutions that undergo rapid transitions to the gel state are prominent carriers in cancer chemotherapy since they localize to the infected site without diffusion to surrounding healthy tissues. In the presence of horseradish peroxidase and H_2_O_2_, curcumin accelerates the di-tyrosine crosslinking gel-forming reaction [15] by increasing silk beta-sheet structures. Moreover, curcumin and silk hydrogels display a synergistic toxic effect on U2Os-osteosarcoma cells (contribution 9).

### 2.10. The MnO_2_/GelMA Composite Hydrogels Improve the ROS Microenvironment of Annulus Fibrosus Cells by Promoting the Antioxidant and Autophagy Through the SIRT1/NRF2 Pathway

Porous, biocompatible intervertebral disk scaffolds composed of hybrid hydrogels containing MnO_2_ and methacrylate gelatin offer a promising alternative for treating degenerated intervertebral disks. They promote autophagy and protect annulus fibrosus cells against oxidative damage by clearing reactive oxygen species, securing a favorable microenvironment for the deteriorated disks (contribution 10).

## 3. Conclusions and Future Directions

Hydrogels represent a tunable and auspicious platform for drug delivery. They may be designed for scaffolds, oral or topical transdermal administration, or via injection. Their characteristics (porosity, elasticity, and swelling) allow cell accumulation, orientation, and proliferation to structures akin to biological tissues. Ongoing research is focusing on more personalized and targeted medicines. Customized compositions are fine-tuned to match the profiles of individual patients, and smart, adaptive receptors dynamically monitor disease progression and adapt release parameters.

Novelties encompass hydrogels that proceed to therapeutic delivery as a response to certain external stimuli such as pH, temperature, concentration of ions, a specific compound, or enzymatic activity. For example, the site-specific cancer medications require pH-triggered formulations suitable for the acidic tumor microenvironment to decrease systemic toxicity. Emerging manufacturing processes, such as 3D bioprinting, permit the fabrication of intricate matrices with accurate regulation over drug position and release rate. Multicomponent hydrogels may include multiple and/or incompatible active ingredients within a single scaffold for combined treatments, while multilayered gel coatings may gradually release different concentrations or protect sensitive therapeutics from environmental factors such as oxidation or humidity.

Looking into the future perspectives, the incorporation of nanomaterials may enhance specificity and accuracy concerning both the release rate and the target substrate. However, there are still challenges that hinder widespread clinical implementation. Every advancing step and adoption of new technology will contribute to addressing limitations such as upscaling and overcoming regulatory issues, relative to the requirement of the absolute reproducibility of structures and activity. Interdisciplinary collaborative research in the fields of chemistry, biology, materials science, and medicine will help accomplish the transition from laboratory experimentations to effective formulations and unlock the full potential of therapeutic hydrogels.
